# Effects of Short-Term Traffic-Related Air Pollution Exposure on Nasal Microbiome in Young Healthy Adults: A Randomized Crossover Controlled Trial

**DOI:** 10.3390/toxics13030180

**Published:** 2025-02-28

**Authors:** Luwei Qin, Jingqi Pan, Demin Feng, Bingqing Yu, Shunyu Li, Xingyu Liu, Yuefei Jin, Shenshen Zhu, Weidong Wu, Wenjie Yang

**Affiliations:** 1 Institute for the Prevention and Control of Non-Communicable Diseases, Henan Center for Disease Control and Prevention, Zhengzhou 450016, China; qinlwxy@163.com; 2 College of Public Health, Zhengzhou University, Zhengzhou 450001, China16650327619@163.com (B.Y.); 18596918848@163.com (S.L.); lxy11192001@163.com (X.L.); jyf201907@zzu.edu.cn (Y.J.); 3 The Third Affiliated Hospital of Zhengzhou University, Zhengzhou 450052, China; yuyuanfenglinna@126.com; 4 The Second Affiliated Hospital of Zhengzhou University, Zhengzhou 450014, China; 5 Henan International Collaborative Laboratory for Health Effects and Intervention of Air Pollution, Xinxiang Medical University, Xinxiang 453003, China

**Keywords:** air pollution, randomized crossover controlled study, nasal microbiome, α-diversity, β-diversity

## Abstract

Traffic-related air pollution (TRAP) remains a concern for public health. However, the exact mechanisms through which TRAP affects the respiratory system are still not fully understood. This study aimed to investigate the nasal microbiome change in healthy adults after short-term exposure to TRAP, contributing to the understanding of the adverse health effects associated with TRAP. A randomized crossover controlled trial was conducted from 9 March to 30 March 2024 among college students aged 19–24 years. Twenty healthy students were recruited through a baseline questionnaire survey and randomly assigned into two groups. One group followed a crowed-testing procedure: the park portion, a three-week washout period, and then the road portion, while the other group experienced the opposite procedure. Both groups were fully exposed to either a park environment or a road environment with high traffic volume. Nasal mucus samples were collected from the participants at the end of the trial, and then 16SrRNA sequencing was performed to analyze the differences in compositional structure and diversity of the nasal microbiome when volunteers were exposed to different levels of TRAP. The α-diversity indices, including the Chao1 index (*p* = 0.0097), observed species index (*p* = 0.0089), and Faith’s PD index (*p* = 0.0255), demonstrated a significant increase in the nasal microbiome of healthy adults following short-term exposure to TRAP. Visualization through a two-dimensional NMDS plot (stress value < 0.2) indicated that nasal bacterial species distribution became richer after TRAP exposure. Furthermore, the relative abundance of nasal *Firmicutes* (*Bacillota*), *Bacteroidota*, and *Actinobacteriota* phyla, especially *Firmicutes* phylum, exhibited a richer distribution after conducting the trial in the road environment with high levels of TRAP, which was shown in the significance test of signature species. Collectively, our study indicates that short-term exposure to TRAP can affect the composition of the nasal microbiota in healthy adults. These findings offer a scientific basis for understanding how TRAP causes respiratory diseases.

## 1. Introduction

Air pollution remains a major public health crisis, with recent estimates from the Global Exposure Mortality Model indicating a substantial increase in the number of global deaths attributed to outdoor air pollution [1]. In 2015, approximately 8.9 million deaths were reported, more than double the previous estimate and surpassing the death toll from cigarette smoking [2]. Outdoor air pollution in many cities around the world, particularly in high-income countries, is primarily caused by emissions from road traffic [3]. These emissions include dust, tailpipe, and non-tailpipe emissions of various pollutants that are harmful to human health and well-being [4]. Nowadays, traffic-related air pollution (TRAP) has emerged as a pressing public health crisis of substantial and escalating proportions. The scope and scale of this issue continue to expand alongside advancements in knowledge and quantification methodologies. TRAP refers to the air pollution generated by modern transportation, including tailpipe emissions from burning coal and oil, as well as non-tailpipe pollutants resulting from friction between vehicles and road surfaces. This type of pollution is a significant concern in urban areas, where high levels of traffic can lead to poor air quality and adverse health effects for residents. It is influenced by a combination of environmental factors such as temperature, humidity, topography, and urban traffic characteristics [5]. Exposure to TRAP can lead to adverse health effects and is a significant risk factor for morbidity on a global scale [6]. Numerous epidemiological and animal studies have demonstrated that TRAP exposure is associated with cardiovascular diseases [7], respiratory diseases [8], and neurological disorders [9]. The inhalation of TRAP leads to oxidative stress and inflammation in the respiratory tract, which serves as a significant trigger for exacerbating chronic respiratory diseases such as asthma, chronic obstructive pulmonary disease, and lung cancer [10].

The ecological niche-specific microbial community acts as a “gatekeeper” during close material interaction between the human body and the external environment, colonizing all regions of the respiratory tract from the nasal cavity to the lungs. It plays a role in the maturation and maintenance of respiratory immunity homeostasis, serving as the guardian of respiratory health [11]. Recent research has demonstrated that the respiratory microbiome serves as a catalyst for the activation of mucosal innate immunity [12], regulates host immune responses [13], and establishes colonization resistance against external harmful microbiota through symbiotic relationships [14]. The anterior nostrils of the respiratory tract are in the closest proximity to the external environment. The nasal bacterial community harbors a rich population of lipophilic skin-colonizing microbiota, such as the genera *Staphylococcus*, *Corynebacterium*, and *Propionibacterium* [15,16], as well as bacteria from other respiratory ecological niches including *Moraxella Fulton* and *Streptococcus* [12,17]. The disruption of nasal microbiome homeostasis is closely linked to a variety of respiratory diseases [18,19]. There is ongoing research into the nasal microbiome with an emphasis on investigating mechanisms of changes and influences on its function and key microbiome-maker species following external environmental exposures that may cause or exacerbate respiratory diseases.

The effects on the respiratory microbiome after exposure to ambient particulate matter have been demonstrated [20]. However, the impact of TRAP on the respiratory microbiome remains unclear. Given the anatomical location of the nasal microbiome, it is one of the first structures to be targeted by TRAP. As a result, TRAP exposure may have a significant impact on its composition and function. While the association of TRAP with the development of respiratory diseases has been established, the potential interactions between the nasal microbiome and pollutants as a cause of the disease have not yet been fully elucidated. In the present study, we conducted a randomized crossover controlled study in young healthy adults to investigate the effects of short-term TRAP exposure on the nasal microbiota, based on a real-world TRAP exposure environment that mimics a population exposed to TRAP during their daily commute. This study provides new insights for understanding of adverse health effects associated with TRAP. It is conducive to further exploration into molecular mechanisms underlying the role and influence of the microbiome in causing or exacerbating respiratory diseases linked with TRAP exposure.

## 2. Materials and Methods

### 2.1. Participants of the Study

A cohort of healthy young adults from Zhengzhou University was recruited as participants through a comprehensive questionnaire survey in March 2024. The survey encompassed baseline information such as gender, age, weight, height, smoking and drinking habits, physical health (with a focus on cardiopulmonary status), lifestyle behaviors (including sleep quality and exercise habits), as well as history of allergies, family hereditary diseases, and medication history. Inclusion criteria for the study subjects were as follows: (1) voluntary participation with informed consent to provide necessary samples for the trial; (2) non-smoking behavior; and (3) absence of infections, cardiopulmonary diseases, respiratory conditions; and no family history of these diseases. Exclusion criteria for the study participants included the following: (1) no alcohol consumption within two weeks prior to the trial; (2) absence of infectious diseases such as cold and flu, along with related medications taken within one month before the trial; (3) no nasal diseases such as allergic rhinitis, sinusitis, nasal polyps, sieve sinusitis, and pteroid sinusitis; (4) no long-term use of medications or health care products; and (5) absence of a disorganized lifestyle including habitual late nights and poor-quality sleep.

A total of 56 questionnaires were collected, and the recruited volunteers underwent screening based on the inclusion and exclusion criteria. Sample size calculations referred to previous similar randomized crossover studies [7]. We screened 24 volunteers for the trial; however, due to voluntary withdrawals, 20 volunteers (10 males and 10 females) aged between 19–24 years old with a BMI range of (19.0–25.9) kg/m^2^ eventually completed all trial procedures. We provided uniform training to all volunteers prior to the commencement of the trial, including explaining the content and arrangement of the trial, precautions, the rights of the subjects, possible harms, and so on. Furthermore, volunteers also received standardized training, which included an explanation of trial content and arrangements, precautions, subject rights, and potential risks. All volunteers signed an informed consent after fully understanding their involvement in the trial. Informed consent was obtained from all subjects involved in the study, and this study was approved by the Ethics Committee of Zhengzhou University (ZZUIRB2024-30).

### 2.2. Study Design

**Figure 1:**
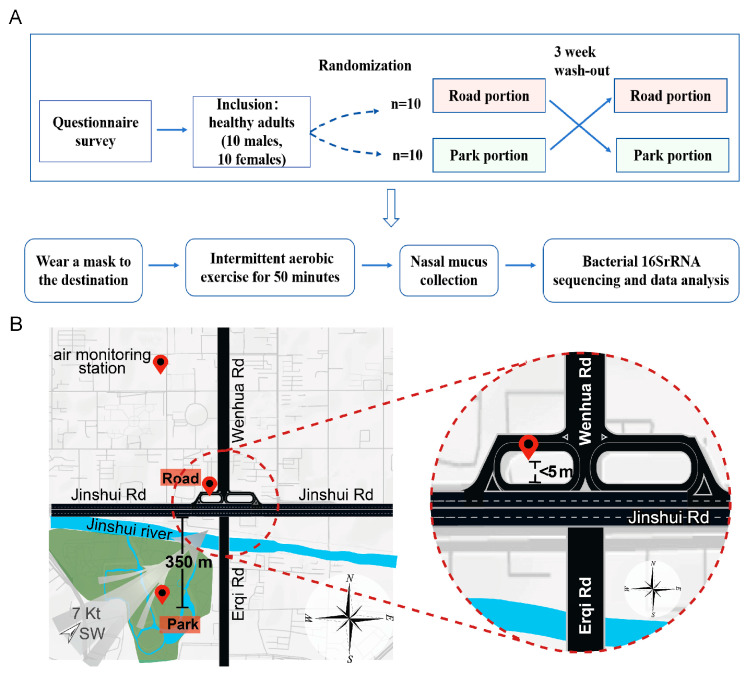
The experimental framework employed in this study. (**A**) Technology roadmap for the experimental design of this study. (**B**) Google Maps was used to identify the exact locations of the road and park environments.

To ensure that each participant experienced distinct exposures and to minimize potential bias, this study employed a randomized crossover experimental design. Specifically, participants were randomly assigned to one of two groups. One group was initially exposed to Treatment A, followed by a rest period, after which they were exposed to Treatment B. Conversely, the other group underwent the same two treatments but in the reverse order. We employed a randomized crossover controlled experimental design to investigate the effects of short-term TRAP exposure on the nasal microbiome of healthy adults. The official experimental period lasted from 9 March 2024 to 30 March 2024. Twenty volunteers were randomly assigned to groups A and B. Group A conducted the initial phase of the experiment in The People’s Park of Zhengzhou as the park group, followed by a three-week washout period, before conducting the second phase in a safe location near the intersection between Jinshui Road, Wenhua Road, and Erqi Road as the road group. The overall trial route for Group A can be summarized as follows: park portion, washout period, road portion. Meanwhile, Group B conducted the trial with a project route consisting of road portion, washout period, and park portion simultaneously. The ambient air quality was good during both phases of the trial, thereby minimizing the influence of the overall ambient air pollution background on the results. To enhance TRAP exposure effects, we scheduled both trial periods between 5:30 pm and 6:30 pm, which coincided with the evening rush hour in Zhengzhou. This time frame has high road traffic volume and intensive vehicle travel. Participants were required to stay within the campus of Zhengzhou University, which is located away from factories and main traffic arteries. The campus has a green space rate of 51%. This arrangement was made in order to ensure consistent baseline environmental exposure levels for the trial subjects prior to the commencement of the trial. In addition, we asked for volunteers to follow a light and moderate diet 48 h before and on the day of the trial, as well as to maintain good sleep quality. On the day of the trial, all volunteers were instructed to wear masks to the trial site and engage in intermittent aerobic exercise simultaneously. The exercise regimen consisted of 1 min of exercise followed by 4 min of rest, repeating this procedure 10 times, which was implemented to control for the impact of exercise intensity on the results. It was anticipated that the mouth and nose of participating volunteers in both road and park sessions would be fully exposed to the environment during the exercise period in order to enhance inhalation of ambient air. Additionally, volunteers were instructed to wear standardized earplugs during exercise to minimize potential confounding effects from noise. Furthermore, it was ensured that volunteers did not leave campus, consume alcohol, become ill, or take medication throughout the entire trial period (Figure 1A).

### 2.3. Exposure Measurements

Hourly average concentrations of air pollutants (PM_2.5_, PM_10_, O_3_, CO, NO_2_, SO_2_), real-time air temperature, and relative humidity data were obtained from the nearest monitoring station located within 1 km of the exposure site via the official China National Environmental Monitoring Center website https://www.cnemc.cn (accessed on 1 April 2024). Information on the wind direction and speed was collected from the official Windy website https://www.windy.com (accessed on 1 April 2024).

In this study, we calculated the distances from the movement locations within the park and road components to their nearest major road. We operated under the assumption that major roads are primary contributors to TRAP exposure environments, and that proximity to these roads is directly proportional to the level of exposure [18]. The park environment is situated at the heart of Zhengzhou People’s Park, surrounded by trees and approximately 350 m from the nearest main road, Jinshui Road. The park is separated from Jinshui Road by the Jinshui River, which contributes to air purification. The road environment is located near an intersection and in a secure position under the Xintong Bridge overpass, less than 5 m away from the busy Jinshui Road with heavy traffic and intense vehicle activity. Both sets of location information can be visualized using Google Maps (Figure 1B).

### 2.4. Nasal Mucus Collection

Nasal mucus samples were collected immediately after each stage of the trial. In detail, we utilized a highly adsorptive strip that has been reported to efficiently collect nasal lining fluid. We utilized a nasal sprayer to administer 100 µL of 0.9% sterile saline solution to both nostrils in order to moisten them, followed by the insertion of the test strip. After a 2-min interval, we removed the test strip and placed it in a sterile 2 mL EP tube, which was then sealed in a foam box filled with ice and promptly transferred to the ultra-low temperature freezer in the laboratory within 1 h.

### 2.5. Extraction and PCR Amplification and Quantification of Total DNA from the Microbiome

The preserved cells were enzymatically digested to fragment the DNA for purification and concentration. Quantification of DNA was performed using Nanodrop, and the molecular size of DNA was determined through 1.2% agarose gel electrophoresis.

PCR amplification specific to the V3-V4 region of bacterial 16SrDNA was carried out with 338F (5′-barcode+ACTCCTACGGGGAGGCAGCA-3′) as the forward primer and 806R (5′- GGACTACHVGGGTWTCTAAT-3′) as the reverse primer. The amplification products were analyzed by 2% agarose gel electrophoresis, followed by excision and recovery of target fragments using magnetic bead purification. Fluorescence quantification of PCR amplification and recovery products was conducted on a microplate reader (BioTek, FLx800, Winooski, VT, USA) using the Quant-iT PicoGreen dsDNA Assay Kit. Subsequently, samples were mixed in a ratio based on fluorescence quantification results and sequencing requirements for each sample.

### 2.6. Sequencing Library Preparation, On-Line Quality Control, and Sequencing

The highly variable V3–V4 region of the bacterial 16S rRNA gene was chosen for sequencing in this study. Sequencing library preparation utilized a TruSeq Nano DNA LT Library Prep Kit (Illumina, San Diego, CA, USA). Subsequently, sequence end repair, addition of bases, and incorporation of sequencing junctions containing index sequences were carried out on the amplification products, with Flow Cells serving as fixed sites for DNA molecules. Following magnetic bead screening to eliminate self-ligated junction fragments, the library system post-junction addition was purified using BECKMAN AMPure XP Beads; the sequencing library template was then enriched and subjected to another round of purification and enrichment. Finally, 2% agarose gel electrophoresis was employed for fragment selection and purification of the library.

Take 1 μL of the library and perform a quality assessment on an Agilent Bioanalyzer machine (Agilent Technologies, Santa Clara, CA, USA) using the Agilent High Sensitivity DNA Kit. Qualified libraries should exhibit a single peak with no junctions. The Promega QuantiFluor Fluorescence Quantification System, in the Quant-iT PicoGreen dsDNA Assay Kit, enables accurate quantification of library concentrations. Libraries meeting the criteria exhibited a concentration of 2 nM or higher.

Qualified libraries were diluted based on gradient (non-repetitive index sequences) and mixed according to the corresponding ratio selected based on the required sequencing volume. Prior to sequencing, mixed libraries were denatured to single-stranded form using NaOH, and the number of libraries was controlled within a range of (15–18) pM as per actual requirements. Bipartite sequencing consisting of (2 × 250) bp was carried out utilizing a NovaSeq sequencer equipped with a NovaSeq 6000 SP Reagent Kit (500 cycles).

### 2.7. Statistical Analysis

The raw data obtained from high-throughput sequencing underwent initial quality filtering and library partitioning, in accordance with the barcode and index information, while simultaneously eliminating barcode sequences. The sequences went through quality control, denoising, splicing, and de-chimerization according to the QIIME2 dada2 analysis process. Taxonomic annotation of the species was performed using the Greengenes2 database after a random rarefaction of obtaining ASV feature sequence length, then conducting taxonomic composition analysis on QIIME2 (2023.7). Additionally, we annotated 16SrRNA gene sequences in the KEGG and MetaCyc databases of PICRUSt2 and conducted a differential analysis of their KEGG metabolic pathways.

Microbial ASV-level α-diversity and β-diversity assays were performed using QIIME2 (2023.7), with subsequent visualization in R. The diversity, richness, evenness, and coverage of microbial communities were characterized using Shannon, Simpson, Faith’s PD, Observed species, Chao1, Pielou’s evenness, and Good’s coverage index. The differences in microbial community composition between groups were explored through principal coordinate’s analysis (PcoA) and nonmetric multidimensional scaling (NMDS) analysis based on Jaccard, Bray-Curtis, unweighted UniFra, and weighted UniFrac distance matrices. Corresponding Adonis and Anosim differential analyses were employed for statistical tests. We also performed orthogonal partial least squares discriminant analysis (OPLS-DA) to assess the overall abundance composition of nasal microbial colonies. Additionally, random forest analysis was utilized to identify prominent nasal bacterial species that significantly contributed to each taxonomic level. ZicoSeq analysis and linear discriminant analysis of effect size (LEfSe) were performed, and paired samples *t*-test or Wilcoxon signed-rank test was also carried out to examine differences in the relative abundance of signature colony species at each bacterial classification level between the road and park sessions. All significance tests were two-sided with α = 0.05, and data normality was confirmed prior to conducting paired *t*-tests. *p*-values were corrected for multiple testing using the Benjamini and Hochberg method for all statistical tests of 16SrRNA data.

## 3. Results

### 3.1. Descriptive Data

The average height was recorded at 172.7 cm, accompanied by a standard deviation of 6.3 cm. Participants’ weight averaged 65.5 kg, with a standard deviation of 7.5 kg, while the mean Body Mass Index (BMI) was calculated to be 21.9 kg/m^2^, with a standard deviation of 1.9 (Table 1). Throughout the study period, no participants reported leaving Zhengzhou or engaging in alcohol consumption, drug use, or experiencing any illness.

### 3.2. Key Air Pollutants and Meteorological Conditions

The ambient relative humidity and temperature in the road and park sessions were similar across both phases of the trial. In addition, air quality in Zhengzhou was assessed as good, with overall air pollutant levels showing no significant differences (Table 2). Wind speed at the trial sites remained consistently at 7 knots (force 3 wind) throughout both periods. Notably, the park section was situated on the upwind side in relation to the road section, as determined by the prevailing wind direction.

### 3.3. Characterization and Diversity of the Nasal Microbiome

**Figure 2:**
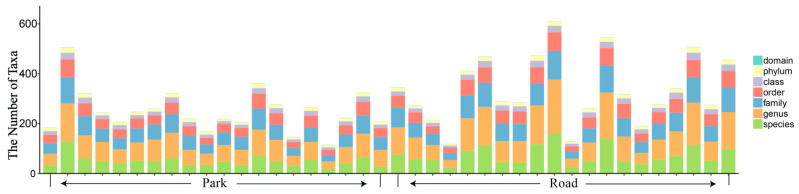
Statistical chart of the number of taxonomic units in each sample.

A total of 3,307,228 high-quality bacterial 16SrRNA sequences were obtained from 80 samples collected from 20 participants. This was achieved through rigorous quality control procedures, which included denoising, splicing, and de-chimerization of primer fragments. All samples underwent testing for bacteria in the prenasal epidermis and nasal mucosa. Each pair of nasal mucus test strips produced between 62,650 and 118,738 sequences, with an average of 87,680.7 sequences. The sequence lengths ranged from 243 bp to 440 bp, with an average length of (417.66 ± 11.24) bp. Sequences were clustered into 9804 amplicon sequence variants (ASVs) following the leveling and filtering of singletons. Figure 2 illustrates the statistical analysis of the number of microbial taxonomic units corresponding to ASV annotations in each sample.

**Figure 3:**
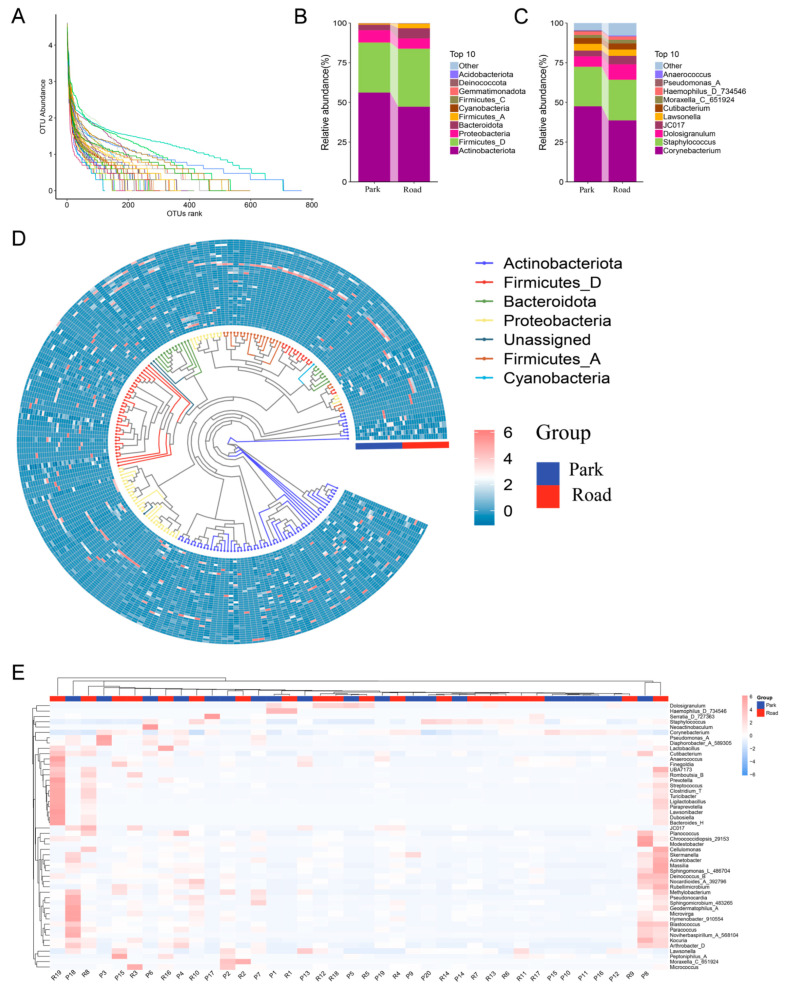
Nasal microbiome distribution of healthy adults. (**A**) Relative species abundance and evenness, depicted through rank abundance (Whittaker) plots. (**B**) Relative abundance distribution of the top ten microbial species on the phylum level. (**C**) Relative abundance distribution of the top ten microbial species on the genus level. (**D**) Evolutionary tree diagram coloring branches according to connected ASV feature sequences on the genus level. (**E**) Clustered heat map of the abundance distribution of the top 50 species on the genus level.

There was no significant difference in the total ASVs of nasal microbiome between participants of the road and park sessions (Figure 3A). The relative abundance of bacterial components in the nasal microbiome of healthy volunteers was assessed at different classification levels from phylum to species, revealing that *Actinobacteriota*, *Firmicutes* (*Bacillota*), *Bacteroidota*, and *Proteobacteria* were the dominant phyla in both road and park portions (Figure 3B). In terms of the relative abundance of the bacterial genus, the nasal cavities of healthy adults were predominantly characterized by *Corynebacteriu*, *Staphylococcus*, and *Dolosigranulum*, as well as a small number of pathogenic bacteria such as *Pseudomona*, *Haemophilus*, and *Moraxella* (Figure 3C). Meanwhile, an evolutionary tree diagram was generated to visualize the phylogenetic relationships of taxonomic units (Figure 3D). This diagram emphasizes the four most abundant phyla previously mentioned in the anterior nares. Furthermore, the species composition heatmaps at the genus level illustrate the distribution of species abundance for each participating volunteer following exposure to varying levels of TRAP (Figure 3E).

### 3.4. Impact of Short-Term Exposure to Traffic-Related Air Pollution on the α-Diversity of the Nasal Microbiome

**Figure 4:**
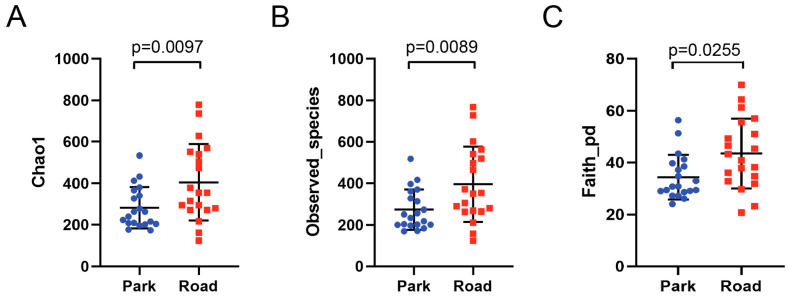
Nasal bacterial α-diversity compared between volunteers from the road section and park section. (**A**–**C**) The ASV-level α-diversity was analyzed for volunteers in the park and road sessions, with richness primarily characterized using Chao1 and Observed species indices, and diversity characterized using Shannon, Simpson, and Faith’s. Error bars show the mean ± SD. Sample size: n = 20 for Park, n = 20 for Road.

The ASV-level α-diversity was analyzed for volunteers in the park and road sessions, with richness primarily characterized using Chao1 and Observed species indices, and diversity characterized using Shannon, Simpson, and Faith’s PD indices. Evenness and coverage were evaluated using Pielou’s evenness index and Good’s coverage index, respectively (Table 3). The Chao1 index for volunteers in the park portion was 282.16 ± 100.03, while the corresponding figure for volunteers in the road session was 404.89 ± 184.17, indicating significant differences between the two sessions (Figure 4A). The number of observed species in the nasal microbiome of participants also significantly increased after TRAP exposure (395.73 ± 181.26) compared to baseline levels (272.98 ± 96.69), with a *p*-value of 0.012 (Figure 4B). These findings suggest that short-term exposure to traffic-related air pollution is associated with an increase in the richness of the nasal microbiome in healthy subjects. Both Shannon’s and Simpson’s index showed a tendency to increase following short-term TRAP exposure, although there was no statistical significance in the results. Notably, Faith’s PD index, which represents the diversity of nasal bacterial communities, was 43.47 ± 13.49 in the road session and 34.32 ± 8.61 in the park session, with a statistically significant difference between the two groups (*p* = 0.016). Therefore, it can be concluded that short-term exposure to TRAP leads to an increased α-diversity of the bacterial microbiome in healthy adults (Figure 4C).

### 3.5. Differences in the Species Composition of the Nasal Microbiome Following Short-Term Exposure to TRAP

**Figure 5:**
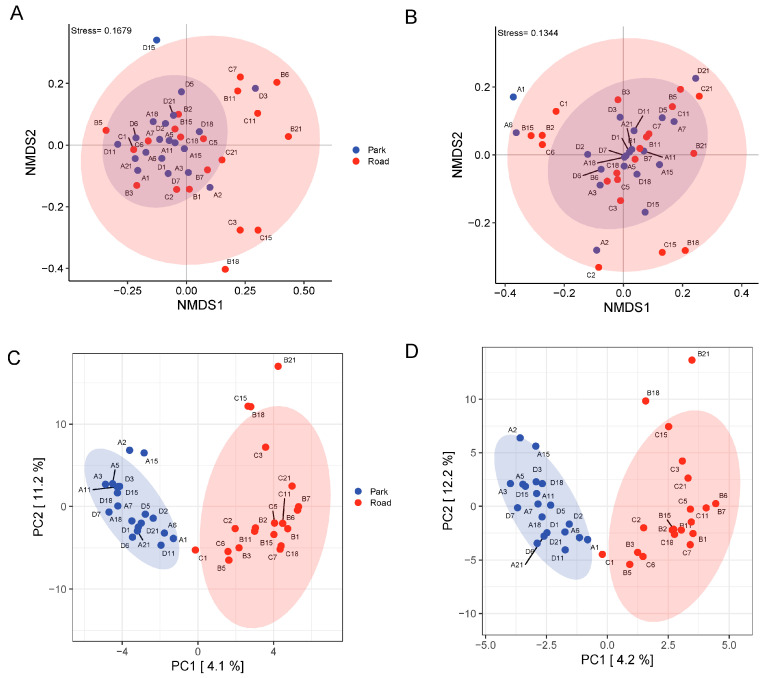
Overall differences in the distribution of nasal microbiome between the road portion and the park portion vol. (**A**) NMDS two-dimensional ranking diagram based on unweighted UniFrac distance matrix. (**B**) NMDS two-dimensional ranking diagram based on weighted UniFrac distance matrix. (**C**) OPLS-DA scores plot for nasal microbes on the family level. (**D**) OPLS-DA scores plot for nasal microbes on the order level. The dots in the graph represent a sample and the colours represent groupings.

In the β-diversity analysis of the samples, four β-diversity distances (Jaccard, Bray-Curtis, unweighted UniFrac, and weighted UniFrac) were computed. The distance matrices obtained from two groups in the road and park portions were subjected to unconstrained ordination for nonmetric multidimensional scaling (NMDS) and principal coordinates analysis (PCoA). It can be observed the value of stress was less than 0.2 in the NMDS analysis of both the unweighted UniFrac and weighted UniFrac distance matrix (Figure 5A,B), indicating a reasonable fit between the two-dimensional distance presentations and actual ecological distances between microbial communities. The overall composition of the nasal microbiome in participants following short-term TRAP exposure appeared to exhibit a richer distribution than that at baseline exposure levels, according to presentation results. However, Anosim differential analysis revealed no statistically significant difference in species composition between these two groups. Additionally, we can visualize the difference in species abundance composition between participants in the road and park portion through OPLS-DA score plots on both the family and order level (Figure 5C,D).

**Figure 6:**
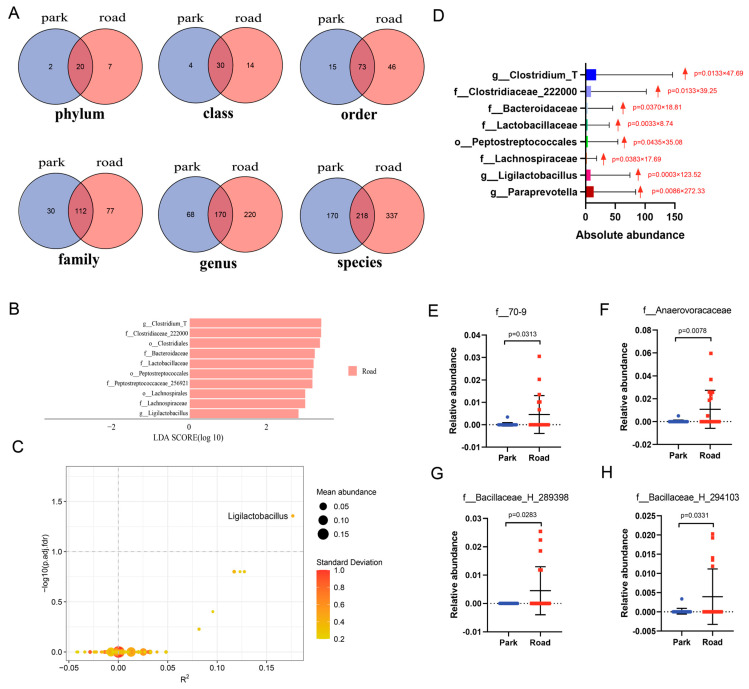
Differences in the nasal signature bacteria between the road portion and park portion volunteers. (**A**) Venn diagrams illustrating the composition of the nasal microbiome on each taxonomic level. (**B**) Histogram depicting the LDA scores of signature strains with significant differences in the road portion. (**C**) Volcano map based on ZicoSeq analysis, with mean abundance representing the average abundance of ASVs and standard deviation indicating the standard deviation of the abundance of ASVs). (**D**) Absolute abundance of the eight most prevalent genera in the road portion, with red arrows denoting a statistically significant increase after exposure to short-term TRAP. (**E**–**H**) Bar graphs displaying the relative abundance of f_70-9, Anaerovoracaceae, Bacillaceae_H_289398, and Bacillaceae_H_29410, with error bars representing mean ± SD. Sample size: n = 20 for Park, n = 20 for Road.

We used Venn diagrams to visually represent the number of shared and unique bacterial species between two groups on each taxonomic level (Figure 6A). Subsequently, ZicoSeq analysis and LEfSe analysis were performed to further investigate the discrepancies in marker bacterial species under different levels of short-term TRAP exposure. It can be observed that the LDA scores of *Clostridiaceae*, *Bacteroidaceae*, *Lactobacillaceae*, *Peptostreptococcales*, and *Lachnospiraceae* in the nasal microbiome of individuals following exposure to high levels of TRAP were all greater than 2 from the results of LEfSe analysis. This elucidated a significant difference in the relative abundance of the above species between the road and park environments, with higher levels observed after high TRAP exposure (Figure 6B,D). *p*-values from ZicoSeq analysis were adjusted for false-positive discovery rate and family-wise error rate, revealing a significant increase in the abundance of genus *Ligilactobacillus* after short-term traffic-related air pollution exposure (*p* = 0.04) as a complement to the results of LEfSe analyses (Figure 6C). In addition, random forest analysis was employed to identify signature bacterial strains contributing more significantly on each taxonomic level, followed by testing their abundances for differences. Table 4 presents the nasal bacterial strains that exhibited differential abundance between the two groups, ranking them in terms of Mean Decrease Accuracy (MDA) on each taxonomic level. It is noteworthy that, in addition to the changes observed in the three previously mentioned phyla, there was a significant increase in the abundance of *Burkholderiales* belonging to *Gammaproteobacteria* in participants from the road portion. Additionally, there was a notable increase in the abundance of all taxonomic levels of *Desulfovibrio* following TRAP exposure. Moreover, a significance test of the top ten marker species in the nasal microbiome ordered by the relative abundance on each taxonomic level indicated significant increases in *Solirubrobacterales* of the *Actinobacterota* phylum and *Anaerovoracaceae* and *Bacillaceae* belonging to the *Firmicutes* phylum after short-term TRAP exposure (Table 5, Figure 6E–H). Thus, short-term TRAP exposure mainly impacted *Firmicutes* (*Bacillota*), *Bacteroidota*, and *Actinobacteriota* phyla of the nasal microbiome in healthy subjects. Among these, species belonging to the *Firmicutes* phylum were found to be most affected.

### 3.6. Predicting the Metabolic Functions of Nasal Microbiota and the Impact of Short-Term TRAP on These Functions

**Figure 7:**
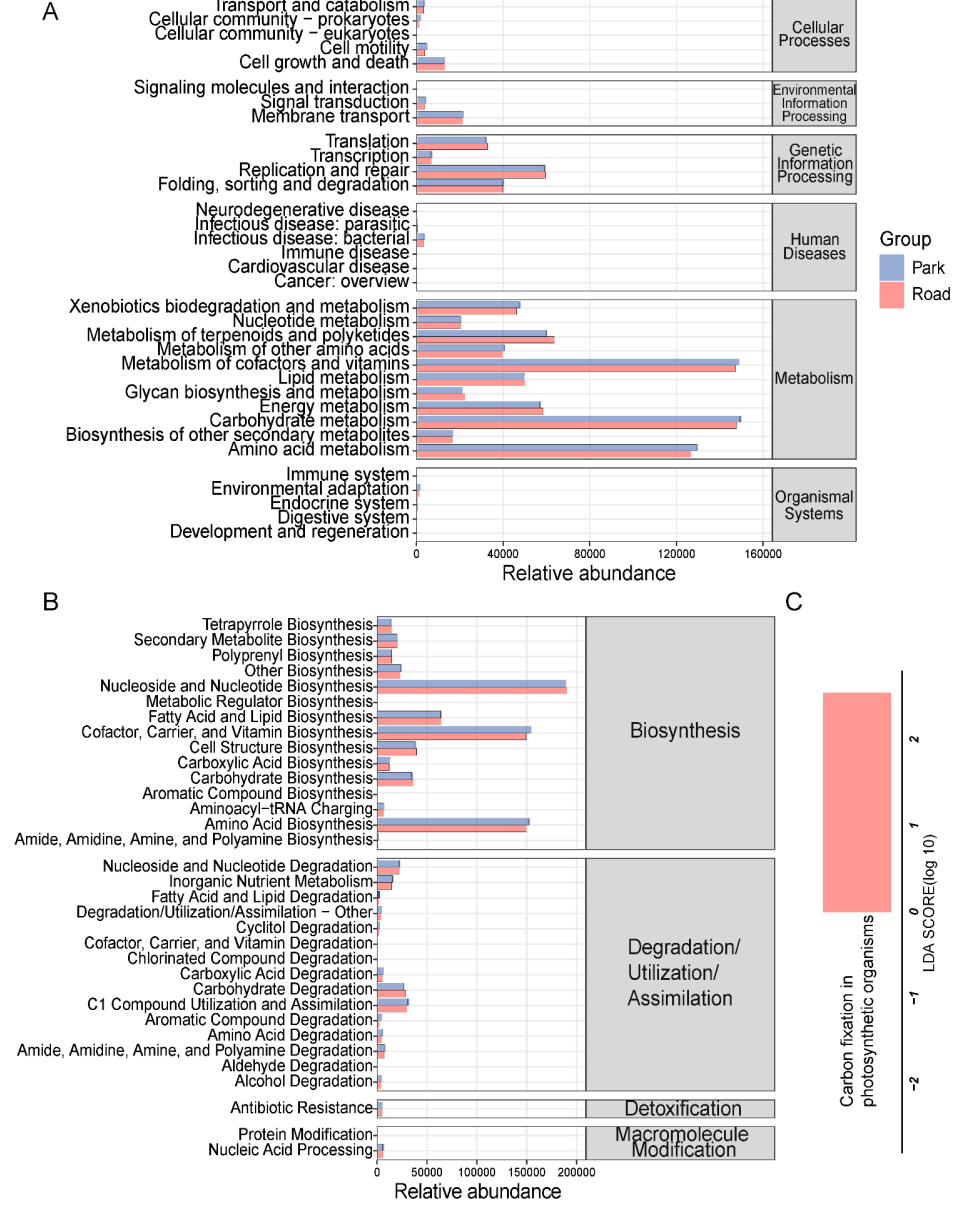
Prediction of metabolic function of the nasal microbiome. (**A**) Predicted KEGG secondary functional pathway abundance map. (**B**) Predicted MetaCyc secondary functional pathway abundance map. (**C**) KEGG metabolic pathway with intergroup differences.

In this study, we quantified the average abundance of the sample microbiome in the secondary functional pathways of the KEGG database for both the road and park portions. The findings revealed that the nasal microbiome primarily functioned in cofactor and vitamin metabolism, carbohydrate metabolism, and amino acid metabolism (Figure 7A). Additionally, at the second categorical level of MetaCyc, our functional analysis demonstrated significant involvement of the nasal microbiome in the biosynthesis of nucleosides, nucleotides, cofactors, carriers, vitamins, and amino acids (Figure 7B). Furthermore, differential analysis conducted on KEGG metabolic pathways indicated that exposure to traffic-related air pollution increased carbon fixation of photosynthetic organisms in the nasal microbiome (Figure 7C). In summary, short-term TRAP exposure may lead to altered metabolic functions of nasal microorganisms.

## 4. Discussion

The major phyla in the nasal microbiome of healthy subjects were *Actinobacteriota*, *Firmicutes* (*Bacillota*), *Bacteroidota*, and *Proteobacteria*, as observed in species classification histograms. Among these, the *Actinobacteriota* and *Firmicutes* phyla were identified as the most dominant nasal phyla, with *Actinobacteriota* accounting for the most predominant proportion of the nasal bacterial community in most individuals while *Firmicutes* predominated in others. The structural features detected were consistent with previous studies [21,22,23]. It is noteworthy that the *Actinobacteriota*, *Firmicutes*, and *Proteobacteria* phyla were previously considered to encompass the majority of detected nasal microbiomes [21]. However, our study revealed a non-negligible contribution of the *Bacteroidota* phylum. Analysis of the top ten relative abundance of species on different bacterial classification levels showed that the *Actinobacteriota* phylum was dominated by *Mycobacteriaceae*, *Propionibacterium*, *Cutibacterium*, and *Corynebacterium*, as well as *Thermoleophilia* and *Micrococcaceae*; the *Firmicutes* phylum was dominated by *Staphylococcus*, *Dolosigranulum*, *Anaerococcus*, *Carnobacteriaceae*, *Lactobacillales*, *Clostridia*, and *Bacilli*; while the *Proteobacteria* phylum was dominated by *Alphaproteobacteria* and *Gammaproteobacteria*, including *Marinilabiliaceae*, *Moraxellaceae*, *Pasteurellaceae*, *Enterobacteriaceae*, *Haemophilus*, and *Pseudomonas*. Additionally, the classes *Cyanobacteriia* and *Gemmatimonadetes* were also detected in healthy adults’ nasal microbiomes. It is well acknowledged that the nasal microbiota consists of harmless commensal bacteria as well as conditionally pathogenic bacteria [11].

The health of the respiratory system is reliant on maintaining a stable state through the interaction of diverse and rich microbiota in the respiratory tract [11]. The nasal cavity serves as the initial body part of contact between external pollutants and the respiratory tract. Once the nasal microbiome homeostasis is disrupted, its function of participating in the maturation and maintenance of respiratory immunity will be affected [24], which increases susceptibility to respiratory-related diseases [25,26]. Previous studies have pointed out that air pollution interferes with the respiratory microbiome [27]. Our investigation focused on the impact of short-term TRAP exposure on α-diversity of the nasal microbiome in healthy adults in terms of three indicators: richness, diversity, and evenness. Significant increases observed in the Chao1 index, observed species, and Faith’s PD index among participants in the road portion suggested that short-term TRAP exposure enhances the richness and diversity of the nasal microbiome. We hypothesize that this may be one of the manifestations of self-regulation of the nasal microbiota in response to acute exposure disturbances. A longitudinal cohort study from birth to early adolescence proposed that exposure to TRAP in early childhood and adolescence leads to an expansion in lower respiratory tract bacterial diversity [20], which is similar to our findings. Due to the limited relevant studies on the effects of traffic-related air pollution on nasal microecology, we focused on several past researches on the effects of PM exposure. Studies on the alteration of mouse lung microbial communities by exposure to different concentrations of PM_2.5_ [28] and the changes in the lung microbiome of broiler chickens exposed to acute TSP [29] exposure produced similar results to our study, demonstrating an enhancement to the α-diversity of the respiratory microbiome. In epidemiologic studies, a study exploring the effects of winter air pollution on healthy youths in northeastern China [30] and a study examining altered pharyngeal microbiome in open-air farmers’ plaza vendors during acute exposure to high levels of PM_2.5_ and PM_10_ [31] both indicated that acute exposure to PM raised respiratory microbiome diversity. Nevertheless, Jacopo Mariani et al. showed a negative association between exposure to PM and nasal microbiota diversity [21]. We postulate that the observed discrepancies in results can be primarily attributed to variations in exposure duration, as their study implemented a one-week exposure period. It is our assumption that there exists an initial increase followed by a subsequent decrease in respiratory microbiome diversity when pollutants accumulate in the respiratory tract over a long period, as also mentioned in the paper written by Wang et al. [32]. Further targeted investigation into this phenomenon is warranted for future research endeavors.

We conducted a comprehensive analysis of the alterations in the overall composition of the nasal bacterial community and the abundance of specific signature species in healthy subjects induced by TRAP exposure. The results from NMDS analysis of β-diversity provide a richer distribution of nasal species in participants in the road portion. Traffic-related air pollution exposure led to an increase in the relative abundance of species associated with the *Firmicutes*, *Bacteroidota*, and *Actinobacteriota* phyla. This is basically consistent with a previous related paper [30], although there is a conflicting finding that the abundance of *Bacteroita* phylum decreased after exposure in that paper. In our study, *Firmicutes* phylum exhibited the most significant changes after short-term TRAP exposure, with its subordinates mainly showing changes in *Clostridiaceae*, *Lactobacillaceae*, *Peptostreptococcales*, *Lachnospiraceae*, and *Anaerobacteria*. Among them, *Lactobacillaceae* and *Lachnospiraceae* have been identified as beneficial bacteria. Some studies have suggested that a decrease in the relative abundance of *Lachnospiraceae* may be linked to the development of asthma [33], while *Lactobacillaceae* may protect the lung tissue by modulating inflammation-hemagglutination interactions [34]. The concurrent increase in the aforementioned two phyla, alongside conditionally pathogenic bacteria such as *Clostridiaceae*, *Peptostreptococcales*, and *Anaerobacteria*, implies an intricate interplay and self-regulation within the nasal mucosal microbiome in healthy adults under short-term TRAP exposure. Within the *Actinobacteria* phylum, a notable rise was primarily observed in their subordinate orders, *Streptomyces* and *Solirubrobacterales*. Notably, there was a significant increase at all taxonomic levels for the *Desulfobacterota* phylum and its subordinate after exposure. It is evident that short-term TRAP exposure disrupts the composition of the nasal microbiome by mainly enhancing its diversity and signature species.

MetaCyc and KEGG are comprehensive databases that integrate genomic, chemical, and systemic functional information [35,36]. The MetaCyc database stands out as the largest metabolic reference database elucidated by experimental data in the life sciences, containing 2722 pathways from 3009 different organisms [37]. We conducted potential functional predictions for the nasal microbiome using both of these databases. Our findings indicate that the nasal microbiome is primarily involved in cofactor and vitamin metabolism, carbohydrate metabolism, and amino acid metabolism, as well as biosynthesis of nucleosides and nucleotides, cofactors, carriers, vitamins, and amino acids. Interestingly, a differential analysis of the mean abundance of nasal ASVs based on the KEGG metabolic-functional pathway revealed an increase in carbon fixation of photosynthetic organisms in the nasal microbiome of healthy subjects after short-term TRAP exposure. Order level analysis demonstrated a significant elevation in *Oscillospirales* belonging to the *Cyanobacteria* phylum within the nasal microbiota in response to TRAP exposure. *Cyanobacteria*, a group of bacteria capable of energy acquisition through oxygen-producing photo-synthesis [38], were nebulized and inhaled by individuals exposed to a TRAP environment, resulting in their accumulation in the nasal cavity [39]. This may partially account for the observed altered metabolic function in the nasal microbiome, as reported in our investigation.

Our results contribute to the understanding of the various effects of TRAP exposure on the human respiratory system and provide potential insights for exploring the associations and mechanisms of the nasal microbiome in respiratory diseases induced by traffic-related air pollution exposure. However, it is important to note that there are limitations to this study. Due to equipment constraints, individual pollutant exposure measurements were not performed, which limits the analytical exploration of the causality between TRAP exposure and changes in nasal microbiota. In the future, a longitudinal study will be conducted to further evaluate the trend of changes in the respiratory microbiome following prolonged exposure to TRAP in healthy populations. We will aim to accurately measure the precise exposure data of specific TRAP pollutants such as organic carbon, nitrogen oxides, carbon monoxide, carbon black, hydrocarbons, and sulfur compounds, so as to establish a “Pollutant Exposure–Respiratory Microbiome Changes–Intermediate Molecules Changes–Endpoint effects” research model. The objective is to examine the interactions and functional effects of the respiratory microbiota in the context of exposure and to compare relevant indices between healthy and diseased populations. This will facilitate the establishment of a causal relationship and the elucidation of specific mechanisms underlying the effects of TRAP exposure on the respiratory microbiota and subsequent relative diseases. Although the subjects in this study were screened using stringent inclusion and exclusion criteria, and efforts were made to control for potential confounding factors as much as possible, several limitations remain. Firstly, it was not feasible to strictly regulate the exposure of subjects to biological factors prior to the commencement of the trial; for instance, the impact of time, influenza, and allergies on nasal flora could not be adequately controlled. Additionally, accurate information regarding the subjects’ personal habits and behaviors was difficult to obtain; some participants may engage in frequent nasal rinsing. Furthermore, the influence of various meteorological factors and components of air pollution on the results remains unknown.

## 5. Conclusions

In conclusion, our study indicates that short-term exposure to TRAP can affect the composition of the nasal microbiota in healthy adults. These findings offer a scientific basis for understanding how TRAP causes respiratory diseases.

## Figures and Tables

**Table 1:** Basic volunteer information.

Variable	Mean (SD)
Gender (male/female)	10/10
Age	21.2 (1.6)
Height (cm)	172.7 (6.3)
Weight (kg)	65.5 (7.5)
BMI (kg/m^2^)	21.9 (1.9)

**Table 2:** Distribution of air pollutants and meteorological conditions during the trial.

	Day	Mean	SD	Min	P_25_	P_50_	P_75_	Max
PM_2.5_ (μg/m^3^)	3.9	46.60	1.14	45.00	45.50	47.00	47.50	48.00
	3.30	25.14	3.08	22.00	22.00	24.00	28.00	29.00
PM_10_ (μg/m^3^)	3.9	95.60	3.78	89.00	92.50	97.00	98.00	98.00
	3.30	131.43	16.79	101.00	123.00	131.00	144.00	151.00
SO_2_ (μg/m^3^)	3.9	6.20	0.45	6.00	6.00	6.00	6.50	7.00
	3.30	5.50	0.84	4.00	4.75	6.00	6.00	6.00
NO_2_ (μg/m^3^)	3.9	19.40	2.07	18.00	18.00	19.00	21.00	23.00
	3.30	13.50	1.64	11.00	12.50	13.50	14.50	16.00
O_3_ (μg/m^3^)	3.9	120.60	4.04	115.00	116.50	122.00	124.00	125.00
	3.30	124.14	5.49	117.00	119.00	124.00	129.00	132.00
CO (mg/m^3^)	3.9	0.40	0.00	0.40	0.40	0.40	0.40	0.40
	3.30	0.50	0.13	0.30	0.40	0.60	0.60	0.60
Temperature (℃)	3.9	21.57	0.79	20.00	21.00	22.00	22.00	22.00
	3.30	23.50	1.23	22.00	22.75	23.00	25.00	25.00
Relative humidity (%)	3.9	15.29	3.55	11.00	13.00	14.00	20.00	20.00
	3.30	21.17	5.49	14.00	14.75	22.50	26.25	27.00

**Table 3:** Distribution of α-diversity indices.

Variable	Mean ± SD	
	Park	Road
Chao1	282.16 ± 100.03	404.89 ± 184.17
Observed species	272.98 ± 96.69	395.73 ± 181.26
Shannon	2.99 ± 0.84	3.35 ± 1.43
Simpson	0.72 ± 0.13	0.76 ± 0.10
Faith’s PD	34.32 ± 8.61	43.47 ± 13.49
Pielou’s evenness	0.37 ± 0.10	0.39 ± 0.10
Good’s coverage (%)	99.95 ± 0.02	99.95 ± 0.02

**Table 4:** The top 10 marker species contributing to each taxonomic level based on random forest analysis, with statistically significant differences observed.

Rank	Taxon	Statistic	*p*-Value	Mean Decrease Accuracy (%)
Phylum	*Patescibacteria*	22.50	0.036	5.75
	*Desulfobacterota_I*	19.00	0.021	2.50
Class	*Saccharimonadia*	16.00	0.024	6.84
	*Desulfovibrionia*	19.00	0.021	4.97
Order	*Saccharimonadales*	16.00	0.024	3.87
	*UBA1381*	0.00	0.036	3.48
	*Streptomycetales_400645*	0.00	0.023	3.32
	*Erysipelotrichales*	29.00	0.026	3.05
	*Burkholderiales_595427*	1.00	0.035	2.26
	*Oscillospirales*	37.00	0.037	1.96
	*Desulfovibrionales*	19.00	0.021	1.35
Family	*Muribaculaceae*	45.00	0.046	3.33
	*70-9*	1.00	0.035	3.01
	*Anaerovoracaceae*	1.00	0.013	2.26
	*Desulfovibrionaceae*	19.00	0.021	2.16
	*Clostridiaceae_222000*	28.00	0.041	1.93
	*Erysipelotrichaceae*	33.00	0.042	1.92
	*Acutalibacteraceae*	7.00	0.042	1.89
Genus	*CAG-41*	0.00	0.036	1.84
	*Desulfovibrio_R_446353*	0.00	0.036	1.49
	*Bacteroides_H*	3.00	0.042	1.00
Species	*Clostridium_T_disporicum_203972*	24.00	0.044	2.87
	*cag-41_sp001941225*	0.00	0.036	2.51
	*Fimenecus_sp000432435*	0.00	0.036	2.35
	*Bacteroides_H_acidifaciens*	1.00	0.035	2.29

**Table 5:** Distribution of differentiated nasal bacterial species.

	Relative Abundance (%)		*p*-Value
	Park	Road	
*f_70-9*	0.017	0.457	0.0313
*Anaerovoracaceae*	0.025	1.078	0.0078
*Bacillaceae_H_289398*	0.000	0.450	0.0283
*Bacillaceae_H_294103*	0.017	0.394	0.0331

## Data Availability

Data are available on reasonable request. Further information can be requested by emailing the principal investigator [wdwu2013@126.com].
